# Analysis of microRNA transcriptome by deep sequencing of small RNA libraries of peripheral blood

**DOI:** 10.1186/1471-2164-11-288

**Published:** 2010-05-07

**Authors:** Candida Vaz, Hafiz M Ahmad, Pratibha Sharma, Rashi Gupta, Lalit Kumar, Ritu Kulshreshtha, Alok Bhattacharya

**Affiliations:** 1 School of Information Technology, Jawaharlal Nehru University, New Delhi, India; 2 School of Life Sciences, Jawaharlal Nehru University, New Delhi, India; 3 Department of Medical Oncology, Institute Rotary Cancer Hospital, All India Institute of Medical Science, New Delhi, India

## Abstract

**Background:**

MicroRNAs are a class of small non-coding RNAs that regulate mRNA expression at the post - transcriptional level and thereby many fundamental biological processes. A number of methods, such as multiplex polymerase chain reaction, microarrays have been developed for profiling levels of known miRNAs. These methods lack the ability to identify novel miRNAs and accurately determine expression at a range of concentrations. Deep or massively parallel sequencing methods are providing suitable platforms for genome wide transcriptome analysis and have the ability to identify novel transcripts.

**Results:**

The results of analysis of small RNA sequences obtained by Solexa technology of normal peripheral blood mononuclear cells, tumor cell lines K562 and HL60 are presented. In general K562 cells displayed overall low level of miRNA population and also low levels of DICER. Some of the highly expressed miRNAs in the leukocytes include several members of the let-7 family, miR-21, 103, 185, 191 and 320a. Comparison of the miRNA profiles of normal versus K562 or HL60 cells revealed a specific set of differentially expressed molecules. Correlation of the miRNA with that of mRNA expression profiles, obtained by microarray, revealed a set of target genes showing inverse correlation with miRNA levels. Relative expression levels of individual miRNAs belonging to a cluster were found to be highly variable. Our computational pipeline also predicted a number of novel miRNAs. Some of the predictions were validated by Real-time RT-PCR and or RNase protection assay. Organization of some of the novel miRNAs in human genome suggests that these may also be part of existing clusters or form new clusters.

**Conclusions:**

We conclude that about 904 miRNAs are expressed in human leukocytes. Out of these 370 are novel miRNAs. We have identified miRNAs that are differentially regulated in normal PBMC with respect to cancer cells, K562 and HL60. Our results suggest that post - transcriptional processes may play a significant role in regulating levels of miRNAs in tumor cells. The study also provides a customized automated computation pipeline for miRNA profiling and identification of novel miRNAs; even those that are missed out by other existing pipelines. The Computational Pipeline is available at the website: http://mirna.jnu.ac.in/deep_sequencing/deep_sequencing.html

## Background

Small non-coding RNAs participate in a variety of processes from cell development and differentiation, stress responses to carcinogenesis by regulating gene expression [1-4]. Regulatory non-coding RNAs have been reported from almost all organisms from bacteria to mammals [5]. Among the various classes of non-coding small RNAs (sRNAs), the most conserved and prominent ones are the microRNAs or miRNAs [for a recent review see, [6]]. Mature miRNA sequences are single stranded, typically 18-24 nucleotides long and encoded as a precursor molecule of about 60-120 nucleotides (in humans) [7]. These precursors are derived from processing pri-miRNA (usually in kilobases) by a ribonuclease, such as DROSHA [8]. Pre-miRNAs are also further cleaved to generate active mature miRNAs with the help of DICER [9]. So far more than 800 miRNAs have been described in human [10]. miRNAs interact with 3'UTR of mRNAs through base pairing and bring about their degradation, destabilization, or repression of translation through RISC, a complex of multiple proteins and miRNA-mRNA adduct [11,12]. Occasionally it can also up regulate gene expression [13].

The expression profiles of miRNAs have been determined in order to understand the role of miRNAs in a specific biological process [14]. The profiles generated revealed altered levels of miRNAs in different systems, such as oncogenesis and development [15,16]. These studies showed strong correlation between specific miRNA expression and phenotype suggesting it can be used as a potential biomarker for diagnosis and prognosis in human cancer [17]. Several groups of miRNAs have been identified that regulate the expression of tumor-associated genes while others seem to hold prognostic value in predicting patient survival. For example, miR-21 is frequently over expressed in various cancers [18]. It is now considered as an oncomiR that acts by down regulating PTEN, a tumor suppressor gene [19]. Similarly let-7 family of miRNAs is frequently down regulated in lung cancers [20]. miR-15a and 16-1 are often deleted in chronic lymphocytic leukemia [21]. These miRNAs target RAS, HMGA2 and BCL2 oncogenes, respectively, thereby regulating tumorigenesis [22-24]. Expression of let-7a, miR-210 and miR-200 clusters was demonstrated to be a strong prognostic marker for lung cancer, breast cancer and ovarian cancer, respectively [25-27]. Many of the fundamental processes are regulated by miRNAs, such as cell differentiation (miR-223, miR-145) [28,29], apoptosis (miR-34, miR-16) [21,30], body patterning (miR-9, miR-196) [31,32], nervous system and muscle development (miR-134, miR-1 and miR-133) [33,34].

A number of different approaches have been used for expression profiling, such as northern analysis [35], cloning [36], real time polymerase chain reaction [37], microarray analysis [38,39] and RNase protection assay [25,40]. These methods are not generally useful for discovery and expression profiling of low-abundance transcripts or yet unidentified novel miRNAs. Recently a number of different platforms have been developed for carrying out large scale parallel sequencing in order to generate genome wide sequences in a short time and reduced cost [41-43]. These have also been applied to analyze transcriptome including sRNA sequences [41]. Generally sequences derived using these platforms can be processed to generate expression profiles of known genes and suitable computational methods can be employed to identify unknown genes. Some of these platforms along with custom computation pipelines have been used to study sRNAs from different systems, such as plants, human embryonic cells and developing chicken embryo [44-46]. All these studies not only generated expression profiles of known miRNAs but also identified a few novel miRNAs. Morin *et al*. used this strategy in combination with RNAfold, MiPred and an in house SVM model to identify novel miRNAs [47]. Though the study identified 104 novel miRNAs there was no attempt to verify these by other experiments. A software pipeline miRDeep has been developed to analyze large-scale sRNA sequencing data and identification of novel miRNAs [48]. Some of the filters used in this approach are highly stringent and it is likely that many novel miRNAs may be missed. Generation of expression profiles and identification of novel miRNAs from deep sequencing is dependent on tools used for analysis. It appears from this discussion that there is a need for development of efficient automated pipelines for analysis of deep sequencing data and setting up validation pipeline for checking the results, as newer algorithms may allow improved profiling and identification of novel miRNAs.

In this manuscript we have profiled sRNA expression from normal human peripheral blood mononuclear cells and two cancer cell lines using Solexa technology and developed automated computation pipelines for analyzing quantitative expression. Our pipelines use statistical analysis of the Solexa sequences for generation of expression profiles and a number of different methods for prediction of novel miRNAs. We believe that these pipelines are highly robust and can be useful for other studies.

## Results

### Sequencing and Annotation of Small RNAs

**Figure 1:**
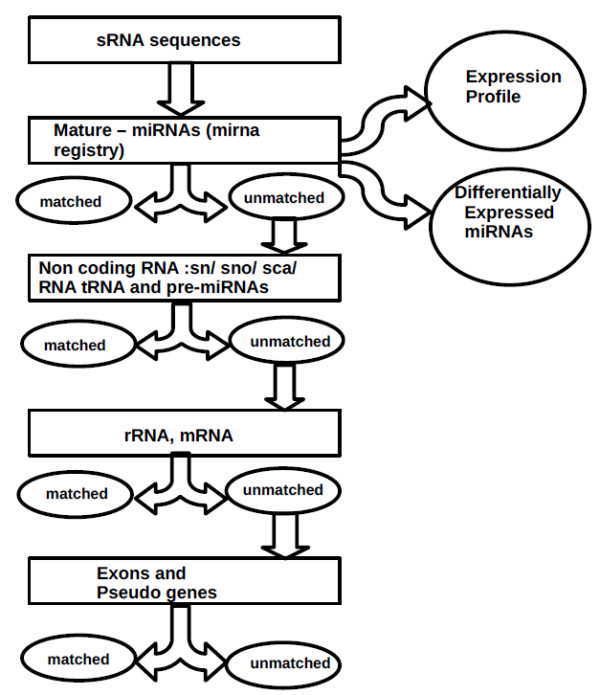
**Flowchart describing the elimination pipeline used to filter out the indicated sequences from the library of sRNA sequences**. The sequences were matched using an "in house" developed fast algorithm. Alignment with maximum of two mismatches was considered as hits. All the hits were removed before the next round of elimination. The databases used in this pipeline were either generated in house or downloaded from publicly available sites as described in "Methods".

**Figure 2:**
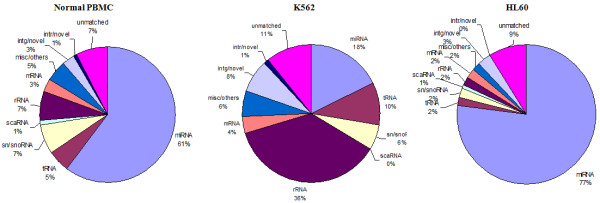
**Frequency of different classes of RNA species present in sRNA libraries**. The sequences obtained from the sRNA libraries were subjected to a series of sequence similarity searches using specific databases (rRNAs, tRNA, sn/snoRNAs, miRNAs, other non-coding RNAs) and the pipeline described in Figure 1. The sequences that did not match with any known sequence were matched against databases of intergenic and intronic regions of the human genome. The pie-charts represent an overview of small RNA gene expression (shown in percentage) in normal PBMC and two cancer cell lines K562 and HL60. Small RNAs belonging to the miRNA family constitute the majority as in normal PBMC (61%) and HL60 (77%) samples. However, in K562 miRNAs constitute only 18% of the sRNA population.

sRNAs were size selected by gel electrophoresis and then sequenced using a Solexa platform (Illumina, USA). The details of sequencing reads of the four samples are given in Additional file 1. The length of an average read was about 33-35 nucleotides. The processing of the raw sequences through computation pipeline is outlined in Figure 1 and described in "Methods". Briefly, the adaptor sequences were first removed from the sequence reads. Only those reads that were greater than 10 nucleotides were considered for further analysis. These sequences were clustered on the basis of sequence similarity and subjected to similarity searches using specific databases (rRNAs, tRNA, sn/snoRNAs, miRNAs, other non-coding RNAs). The reads that did not match known RNA sequences were checked to see if these were encoded by intergenic, intronic or exonic regions of the human genome (Figure 2).

Sequencing reads derived from mRNAs and rRNAs made up about 2-4 and 2-13% of the total reads of all the samples, respectively except that from K562 cells. Since mRNAs and rRNAs are likely to have been derived by degradation it appears that the degradation was minimal in these samples. The sequencing reads derived from miRNAs were estimated to be about 60-80%, similar to some of the other studies on sRNAs [47]. The result from K562 was quite different. Only about 18% of total sRNA population was found to be derived from miRNA. Low level of miRNAs in K562 cells is unlikely to be due to artifact as we got similar values in two independent experiments (data not shown). Out of a total of 904 miRNAs (718 major and 186 star sequences) present in miRBase version 14, 534 miRNAs were detected in at least one of the four samples sequenced [49]. A total of 370 miRNAs were not detected in any of the samples.

### MicroRNA expression patterns

**Figure 3:**
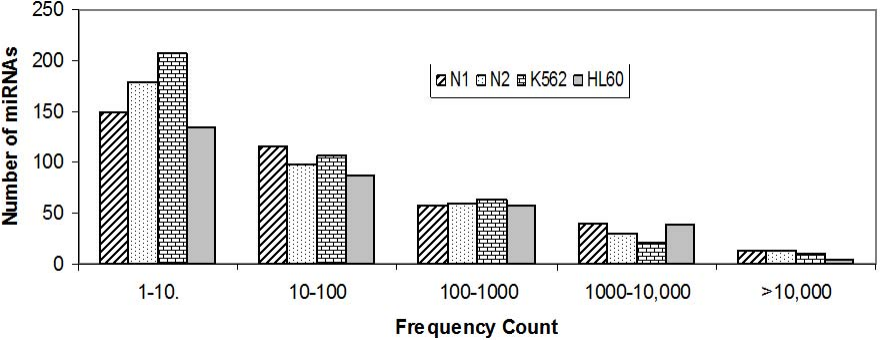
**Overall level of expression of known miRNAs**. The distribution of known miRNA levels with respect to number of miRNAs is shown. Numbers of sequence reads are taken as miRNA levels and the values are represented in the form of range of values. The expression levels of the miRNAs span up to five orders of magnitude.

**Figure 4:**
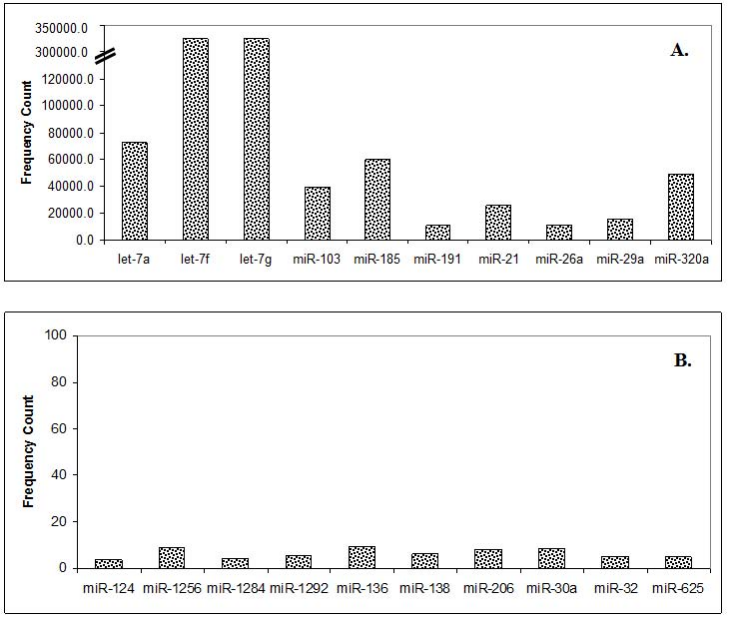
**The abundance of selected miRNAs in human normal PBMC**. The numbers of reads were used as expression level of respective miRNAs. [A]. Some of the highly expressing miRNAs (> 10,000 counts). [B]. Some of the low expressing miRNAs (< 10 counts).

Absolute sequence reads were transformed into transcript abundance by first normalizing the data in 'transcripts per million (TPM)' for each library (see "Methods"). The expression levels ranged from less than 10 to more than 100,000 counts (Figure 3, refer Additional file 1). Thus the sequencing data revealed a wide range of expression levels spanning five orders of magnitude. Several members of the let-7 family, 103, 185 and 320a were observed as some of the highly expressed miRNAs (Figure 4). These miRNAs were also reported as highly abundant miRNAs in peripheral blood mononuclear cells using "Taqman microRNA assay" [50]. While the members of let-7 family, let-7a, f and g represented about 77% of the total miRNA counts, a number of miRNAs with less than 10 counts including singletons were also noticed (Additional file 1). It is not clear if the singletons represent true transcript or noise of the system. miR-219-5p was one of the singletons found in all the four samples suggesting that such sequences may not be due to experimental noise or a chance event. Star sequences of many miRNAs (20% of all known miRNAs) were also observed mostly at low abundance level. Some of the highly abundant star sequences observed by us were miR-106-b* (N1, TPM- 288.28); miR-17* (N1, TPM-1321.41); miR-92a-1* (N1, TPM- 942.78), miR- 25* (N1, TPM-265.44), and miR-374a* (N1, TPM-201.34). The results suggest that sRNA sequencing is a good approach for studying miRNA* abundance.

### Expression patterns of miRNA clusters

miRNAs are often present in gene clusters and it is generally believed that individual miRNAs are generated from common polycistronic transcript by post - transcriptional processing [51,52]. Since the genes in clusters are co-regulated and co-transcribed it is expected that the levels of these miRNAs are likely to be similar. This was investigated by checking the relative levels of individual miRNAs present in clusters using the expression patterns derived from deep sequencing. A total of 265 miRNA genes organized in 68 clusters were studied. As the sequencing reads represent mainly mature miRNAs and not individual precursors, it was not possible to analyze some of the clusters, such as those containing miR-15a, 16-1 and 15b, 16-2 that encode different precursors but the same mature miRNA. Furthermore, 19 clusters were also excluded from analysis as the miRNAs encoded in these clusters were either not expressed or expressed below 1 TPM based on our data. Therefore in this study results from analysis of only 20 clusters are shown (Additional file 2).

**Figure 5:**
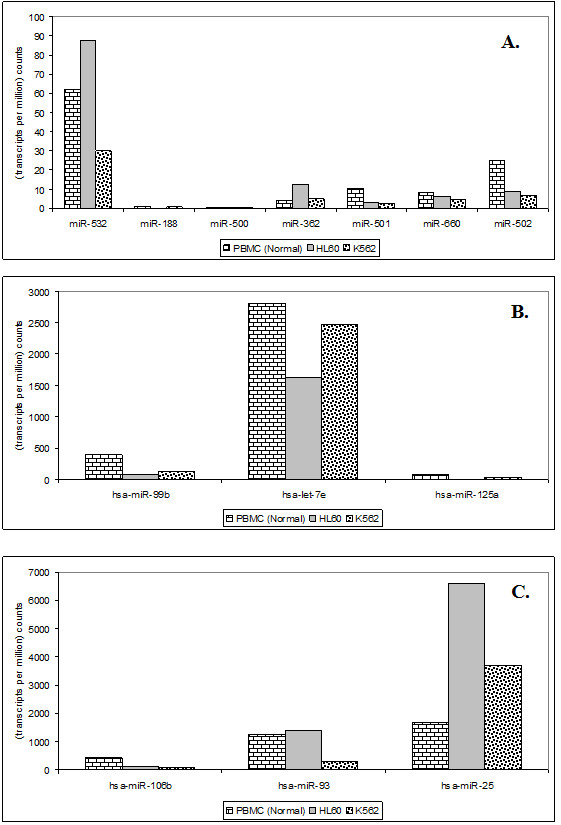
**Differential expression of individual miRNAs present in the same cluster in different datasets**. Here TPM (transcript per million) is used as a measure of expression. [A] miRNAs belonging to cluster miR-532, [B] cluster miR-99b and [C] cluster miR-106b in normal PBMC, K562 and HL60 cell lines. A large variation in expression levels of different miRNAs present within the same cluster is observed.

The level of expression of different miRNAs in many clusters displayed variable expression as exemplified by the cluster containing miR-532 and 99b (Figure 5). In some cases the variation observed within a cluster was as much as 500 fold and the miRNA that showed highest level of expression was not the one closest to the transcription start site. For example, miR-25 showed highest expression though it was farthest from the start site in miR-106b cluster (Figure 5). This is likely to be due to variation in processing mechanisms at post - transcriptional level as has been seen in case of p53-mediated alteration in processing some of the miRNAs, such as miR-16-1 [53]. Overall the results suggest that the levels of miRNAs in any cell may be regulated by a number of different processes and mechanistic details of many of these are not yet known.

### Differentially Regulated miRNAs in K562 and HL60

**Figure 6:**
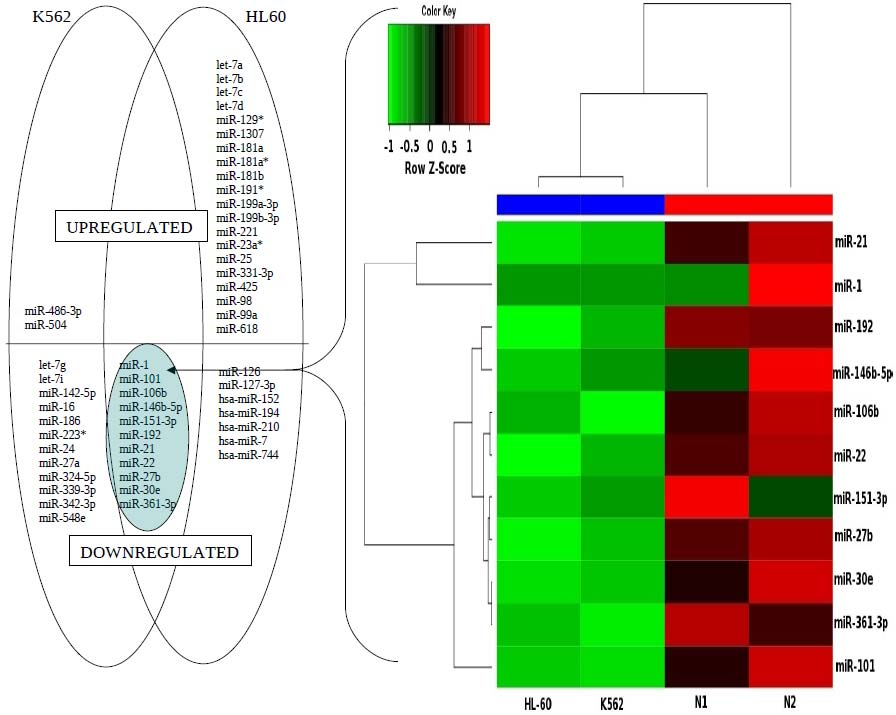
**Differentially regulated known miRNAs**. Up regulated/down regulated miRNAs are represented in the form of Venn diagrams. A subset of miRNAs that are differentially regulated but common in both cell lines as compared to normal PBMC is in the overlapped area and their expression levels can be seen in the heat map. Heat map of some of the differentially regulated known miRNAs with respect to datasets from normal PBMC and cancer cell lines K562 and HL60 is shown as an inset.

**Figure 7:**
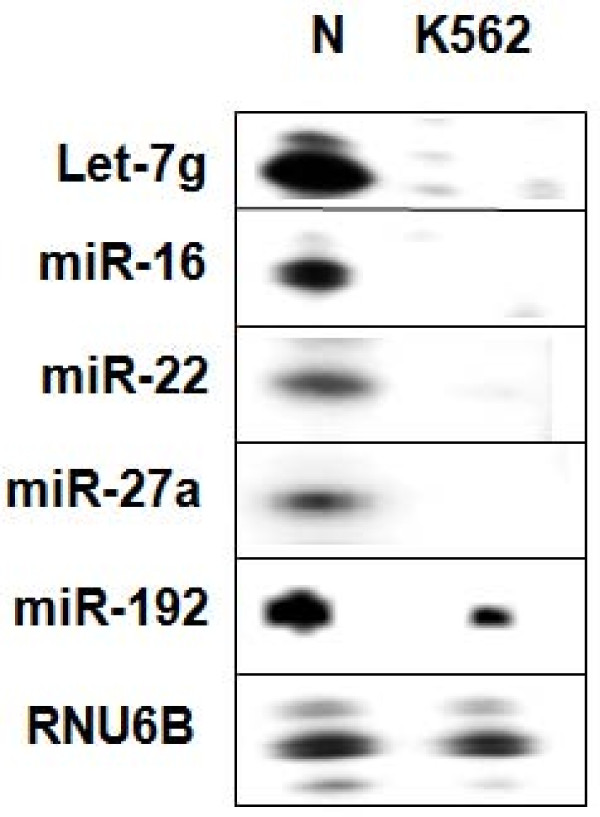
**Expression levels of some of the known miRNAs determined by RNase protection assay**. The relative expression levels of some of the differentially regulated miRNAs were determined using RPA. Briefly, total RNA from indicated cells was incubated with a labelled probe specific for a given miRNA and eventually treated with ribonuclease as described in the "Methods". The protected fragments, suggesting presence of specific transcripts, were first separated on 12% urea PAGE and then visualized by phosphorimager. Loading control was transcripts corresponding to RNU6B visualized using RPA.

Microarray based methods have been used extensively to profile expression levels of annotated genes at genomic scale. These methods cannot be used satisfactorily for analyzing expression levels of unknown transcripts. Deep sequencing of cellular RNAs is an alternative approach for deciphering expression profiles of genes [46,54]. Though a few reports describing analysis of differentially expressed miRNAs deciphered by deep sequencing are available, there is no study yet on human PBMC. Moreover, there is no standardized tool available for profiling miRNA expression using deep sequencing of sRNA [47,55]. Therefore we have studied normal peripheral blood mononuclear cells from two different individuals and cancer cells of myeloid lineage, K562 (chronic myelocytic leukemia) and HL60 (acute promyelocytic leukemia) by deep sequencing of sRNAs followed by analysis of the sequences using a custom designed computation pipeline. We used intersection of two methods for finding differentially expressed genes; a) SAM (significance analysis of microarrays) that allows for the control of false detection rate (FDR) and b) fold change, that is, miRNAs that showed more than 2.5 fold differences when compared to both normal samples. A list of differentially regulated miRNAs is shown (Figure 6). Interestingly, miR-1, 101, 106b, 146b-5p, 151-3p, 192, 21, 22, 27b, 30e and 361-3p displayed low expression in both the cell lines indicating a common role in leukemia genesis or progression (Figure 6, see inset). In K562 most of the miRNAs were down regulated except miR-486-3p and miR-504. The expression patterns derived from sRNA sequences were validated by RNase protection assay (RPA) which allowed both qualitative and quantitative analysis of RNA levels [25,56]. Down regulation of expression of some of the miRNAs, such as miR-16, 22, 27a, 192 and let-7g in CML cell line K562 was observed using RPA assay (Figure 7). RNU6B was used as a loading control, for all the samples. We have also used quantitative RT-PCR for validating some of the results derived by deep sequencing and RPA. Fold differences in the expression of some of the differentially expressed genes in normal and K562 cells (miR-22 and miR-27a) using all three methods were found to be comparable indicating quantitative nature of these approaches (Additional file 3).

While miR-21 was found to be one of the down regulated miRNAs in our study, it is expressed at high level in all the cancers tested till date indicating that miRNA expression is likely to be cell type and context dependent [18]. Reduced expression of some of the miRNAs, such as miR-16, 151 and 142 was previously reported in CML [57-59]. Up regulation of the polycistronic miR-17-92 cluster in CD34+ cells of CML patients was initially described by Venturini *et al*., 2007 and later was challenged by Agirre *et al*., 2008 [59,60]. According to the latter group there is no significant increase in the expression of this cluster in CML cells. We too did not observe any induction of this cluster in K562 cell line in agreement with the latter group.

**Figure 8:**
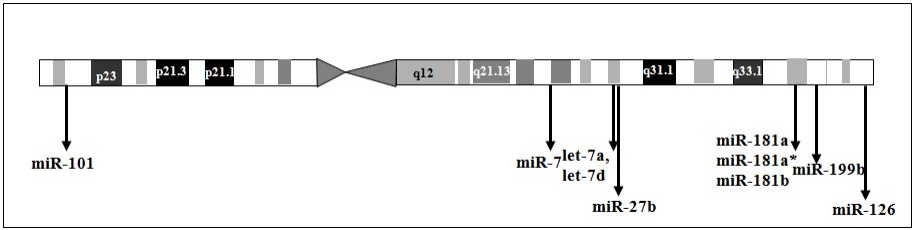
**A map of chromosome 9 showing locations of the differentially expressed HL60 miRNAs**. The differentially expressed HL60 miRNAs were mapped to chromosomes based on the coordinates (GRCh37) available on miRBase version 14. The chromosome 9 is shown here as most of the miRNAs mapped to this chromosome.

A significant number of differentially expressed miRNAs of HL60 (downregulated-miR-101, 126, 27b, 7; up regulated- let-7a, let-7d, miR-181a, -181a*, -181b and miR-199b) were mapped to chromosome 9 (Figure 8). Differential expression of miR-181a family (miR-181a, 181a* and 181b) was previously reported [61]. Some of the differentially regulated miRNAs of K562, such as miR-101, miR-27b and miR-24 were also encoded by chromosome 9. The genomic locations of some of these miRNAs were distant from the ABL gene locus. Alterations in chromosome 9 are associated with a large number of diseases, particularly cancer [62]. Therefore, there may be a link between alterations in chromosome 9 and differential expression of miRNAs.

### Predicted targets of differentially regulated miRNAs

Identification of putative targets may help to understand the biological role of the differentially regulated miRNAs. In this study two different approaches were used for target identification. In the first approach a number of different software tools were employed for target prediction and in the second approach mRNA expression profiles were generated and the genes that showed inverse correlation with miRNA expression were identified. The second approach is based on a number of observations where target mRNA degradation by miRNAs were reported [63,64]. For computational prediction stringent criteria, that is, only those genes that are predicted to be target of specific miRNAs by at least five of the eleven established target prediction programs (DIANA-microT, MicroInspector, miRanda, MirTarget2, miTarget, NBmiRTar, PicTar, PITA, RNA22, RNAhybrid, and TargetScan/TargertScanS) integrated by miRecords and also showed expression levels that are inversely correlated with the specific miRNA levels were considered [65]. Putative targets of differentially regulated miRNAs are shown in Additional file 4 (K562) and Additional file 5 (HL60). Genes targeted by three or more miRNAs are shown in Table 1. Genes that are targets of multiple miRNAs are likely to be tightly regulated and may show graded response on the basis of expression of different miRNAs [66]. MEIS2, up regulated in K562, was found to be the target of up to five different miRNAs, down regulated in both K562 and HL60. MEIS2 has been previously reported to be important for myeloid leukemogenesis [67]. Similarly, SMAD7 a down regulated gene in HL60 is predicted to be a target of three miRNAs, up regulated in HL60. It is known that SMAD7 regulates SMAD and mitogen activated kinase (MAPKs) signaling and controls erythroid and megakaryocytic differentiation of erythroleukemia cells [68]. TRIB2, multiply targeted by three up regulated miRNAs in HL60 has been reported to be present at low levels in AML [69].

**Table 1:** List of Genes targeted by 3 or more differentially regulated miRNAs in K562 and HL60.

Genes targeted by 3 or more downregulated miRNAs in K562		Genes targeted by 3 or more downregulated miRNAs in HL60		Genes targeted by 3 or more upregulated miRNAs in HL60	
**ABCC5**	let-7g, let-7i, miR-101	**BCL11A**	miR-1, miR-21, miR-152	**ARID3B**	let-7a, let-7c, let-7d, miR-98
**ACVR2B**	miR-16, miR-101, miR-186,	**EDEM3**	miR-27b, miR-30e, miR-101	**ARRDC3**	miR-25, miR-181a, miR-181b
**ADAMTS3**	miR-16, miR-30e, miR-146b-5p	**GALNT7**	miR-7, miR-27b, miR-30e	**BAZ2A**	miR-25, miR-99a, miR-181a
**ARRDC4**	miR-27a, miR-27b, let-7i, let-7g	**MEIS2**	miR-27b, miR-30e, miR-101, miR-194	**CCR7**	let-7a, let-7b, let-7c, let-7d
**BZW2**	let-7g, let-7i, miR-101	**PHF6**	miR-1, miR-30e, miR-101, miR-106b	**CD200R1**	let-7a, let-7b, let-7c, let-7d, miR-98
**CDC25A**	let-7g, let-7i, miR-16, miR-21, miR-339-3p	**PTGFRN**	miR-30e, miR-106b, miR-146b-5p	**CPEB2**	let-7a, let-7b, let-7c, let-7d, miR-25, miR-98
**CIT**	miR-27a, miR-27b, miR-30e, miR-142-5p	**SATB2**	miR-22, miR-27b, miR-30e	**CPEB4**	miR-25, miR-181a, miR-181b
**CLCN3**	miR-1, miR-101, miR-27a, miR-27b			**DUSP16**	let-7a, let-7b, let-7c, miR-98
**CLDN12**	let-7g, let-7i, miR-16			**EGR3**	let-7a, let-7b, let-7c, let-7d, miR-98, miR-181a
**CPEB1**	let-7g, let-7i, miR-1, miR-22			**EPHA4**	let-7a, let-7b, let-7c, miR-98, miR-181a
**DCBLD2**	miR-16, miR-24, miR-101			**FOS**	miR-181a, miR-181b, miR-221
**E2F7**	miR-16, miR-27a, miR-27b			**GCNT4**	let-7a, let-7b, let-7c, let-7d, miR-98
**GALNT7**	miR-27a, miR-27b, miR-30e			**GPX7**	let-7a, let-7b, let-7c
**HIC2**	let-7g, let-7i, miR-24, miR-30e, miR-146b-5p			**IGF2BP2**	let-7a, let-7b, let-7c, miR-98, miR-181a
**IGF2BP3**	let-7g, let-7i, miR-142-5p			**ITGB3**	let-7a, let-7b, let-7c, miR-98
**LIN28B**	let-7g, let-7i, miR-30e			**KIAA1539**	let-7a, let-7b, let-7c, let-7d, miR-98
**MEIS2**	let-7i, miR-27a, miR-27b, miR-30e, miR-101			**KLHL24**	let-7a, let-7b, let-7c, miR-98, miR-618
**NEDD4**	miR-27a, miR-27b, miR-30e			**LRIG1**	let-7a, let-7b, let-7c, let-7d, miR-98, miR-425
**PDIK1L**	miR-1, miR-16, miR-22, miR-142-5p			**MAP3K3**	let-7b, miR-181a, miR-181b
**PHF6**	miR-1, miR-30e, miR-106b, miR-186			**MYO1F**	let-7a, let-7b, let-7c, let-7d
**PTGFRN**	miR-30e, miR-106, miR-146b-5p			**PPP1R16B**	let-7a, let-7b, let-7c, let-7d, miR-98
**RELN**	miR-16, miR-27a, miR-27b			**PRKCE**	miR-25, miR-181a, miR-181b
**SATB2**	miR-16, miR-22, miR-27a, miR-27b, miR-30e			**RAB11FIP4**	let-7a, let-7b, let-7c, let-7d, miR-98
**SGMS1**	miR-27a, miR-27b, miR-106b, miR-142-5p			**SLAMF6**	let-7a, let-7b, let-7c, let-7d, miR-98
**SPRED1**	miR-1, miR-16, miR-101			**SLC30A4**	let-7a, let-7b, let-7c, miR-98
**STRBP**	let-7g, let-7i, miR-146b-5p			**SLC35D2**	let-7a, let-7b, let-7c, let-7d, miR-98
**WEE 1**	miR-16, miR-27a, miR-106b			**SLC4A4**	let-7a, let-7b, let-7c, let-7d
				**SMAD7**	miR-25, miR-181a, miR-181b
				**SNN**	let-7a, let-7b, let-7c, let-7d,
					miR-25, miR-98, miR-181a
				**STK40**	let-7a, let-7b, let-7c, let-7d,
					miR-98
				**SYT11**	let-7a, let-7b, let-7c, let-7d
				**TMEM2**	let-7a, let-7b, let-7c, let-7d
				**TRIB2**	let-7a, let-7b, let-7c, miR-98
				**UTRN**	let-7a, let-7b, let-7c, let-7d, miR-98

### Expression of intronic miRNAs

Many intron-encoded miRNAs are processed in DROSHA independent way (miRtrons) [70]. We have also analysed expression levels of differentially regulated intronic miRNAs. In order to check if differential expression of intronic miRNAs is due to transcriptional or post - transcriptional mechanisms, the levels of intronic miRNAs were compared with that of the host transcripts. Comparative analysis revealed positive correlations with respect to 8 out of 14 intronic miRNA - mRNA pairs (Table 2). Unlike miRNAs in clusters, the expression of some of the intronic miRNAs may be transcriptionally controlled.

**Table 2:** Correlation of expression patterns in A) K562 and B) HL60 cancer lines between differentially regulated intronic miRNAs and their host genes.

A)	K562	
**miRNA**	**Host Transcript**	**miRNA:Host transcript Status**
miR-342	EVL	Downregulated
miR-548e	SHOC2	Downregulated
miR-486	ANK1	Upregulated
		

**B)**	**HL60**	

**miRNA**	**Host Transcript**	**miRNA:Host transcript Status**
miR-22	C17orf91	Downregulated
miR-151	PTK2	Downregulated
miR-199b	DNM1	Upregulated
miR-25	MCM7	Upregulated
miR-618	LIN7A	Upregulated

### miRNA Biogenesis Machinery

The analysis of microarray data revealed induction in the expression of some of the miRNA biogenesis genes (RNASEN, DGCR8, XPO5, RAN) in K562 cell line (Table 3). However, DICER1 was found to be down regulated (1.79 fold). This would result in a likely increase in the pre-miRNA population in cytosol but decrease in accumulation of mature miRNAs. This is consistent with the observation that K562 cells contain relatively less amount of miRNAs and that 23 out of 25 differentially expressed miRNAs are down regulated (see Figure 6). This observation was not due to artifact as two independent sequencing of sRNAs from K562 cells gave similar results (data not shown here). Low DICER and let-7 levels were also observed in lung adenocarcinoma [20]. In contrast, a higher level of DICER1 in prostate adenocarcinoma accounted for up regulation of 39 of 45 differentially expressed miRNAs [71]. In HL60 some of the components of biogenesis machinery (RNASEN and XPO5) were found to be up regulated.

**Table 3:** Altered levels of miRNA biogenesis and miRISC components in K562 and HL60.

Protein	Function	K562/Normal (Fold Change)	HL60/Normal (Fold Change)
DROSHA (RNASEN)	PrimiRNA processing	2.238954	2.017825
DGCR8	PrimiRNA processing	2.078018	Unchanged
XPO5	Exporting premiRNA	2.669646	2.199101
RAN	Exporting premiRNA	1.832214	Unchanged
DICER1	PremiRNA processing	-1.79971	Unchanged

### Identification of Novel MicroRNA genes

**Figure 9:**
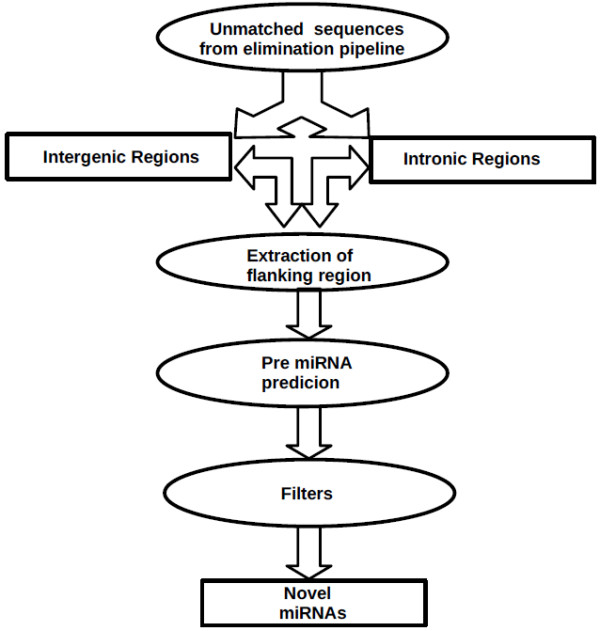
**Flowchart describing the computational pipeline used for prediction of novel miRNAs**. The sequencing reads that did not match with any of the databases of elimination pipeline, but matched with the human intergenic and intronic sequences, were extracted along with flanking sequences from human genome. These were then analysed by a number of miRNA precursor prediction algorithms and the hits were further analysed by a set of filters as described. The final output of the pipeline gives a list of novel miRNAs.

**Figure 10:**
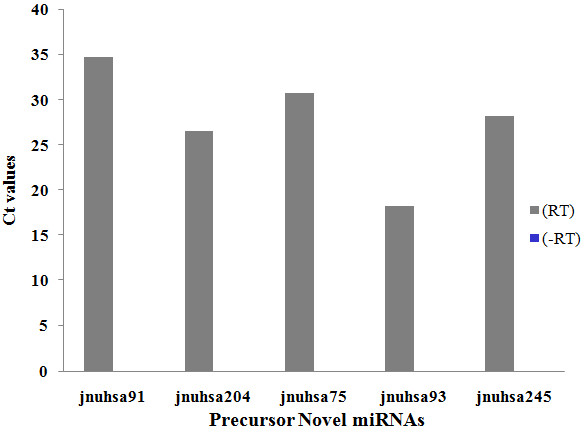
**Detection of precursor novel miRNAs through Real-time PCR**. Real-time PCR confirmation of the precursors of novel miRNAs predicted through CID, CSHMM, MiPred tools. A no-RT-PCR reaction is used as negative control.

**Figure 11:**
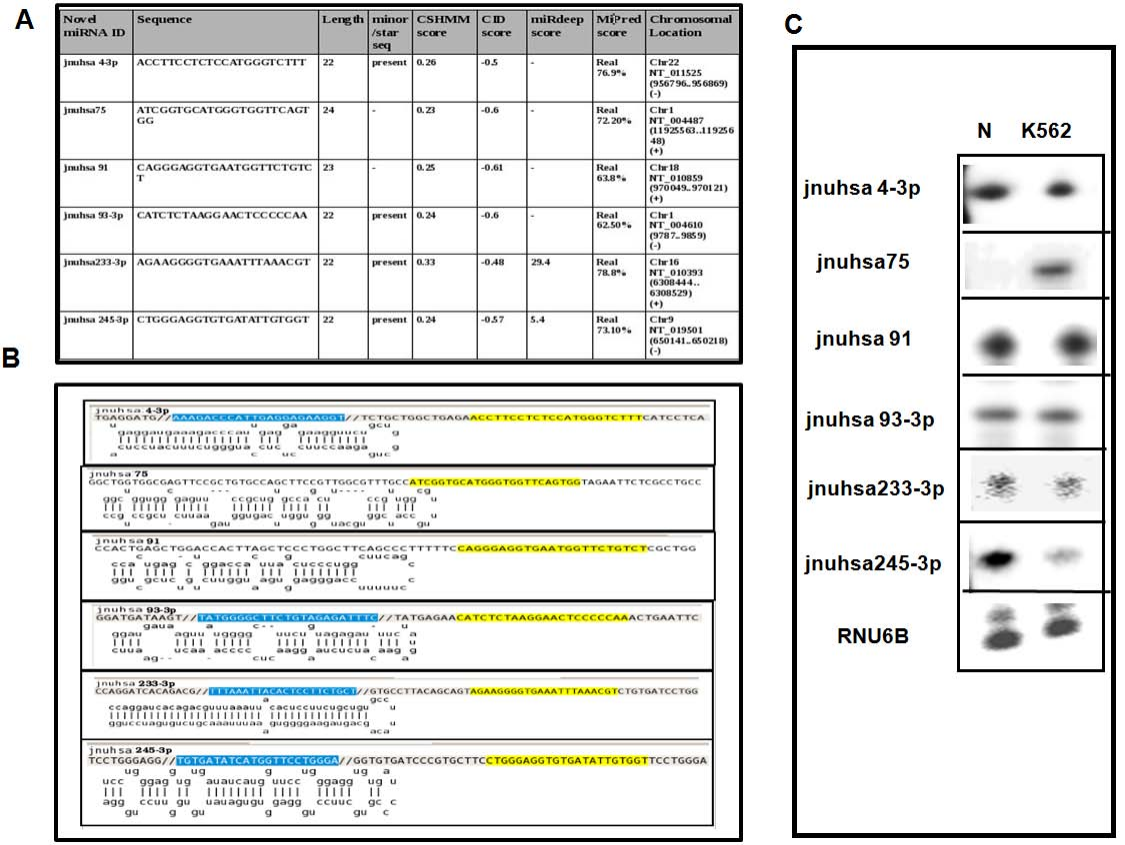
**Predicted novel miRNAs**. A. A partial list of novel miRNAs predicted from deep sequencing data is displayed along with chromosomal location and the scores from different prediction tools. B. The precursor sequence and the secondary structure of the novel miRNAs. The highlighted regions in blue and yellow colour indicate the presence of 5p and 3p mature miRNA sequences, respectively. Note that the sequenced mature putative miRNAs map to the stem part of the structure. C. The expressions of these miRNAs were independently validated by RPA. RPA was carried out as described in the legend for Figure 7 using total RNA from normal PBMC and K562 cell lines. The phosphor imager images are shown. RNU6B transcripts were used as a control. Some of the miRNA star sequences were also detected. The brightness/contrast have been changed to normalize the signals across different probes.

**Figure 12:**
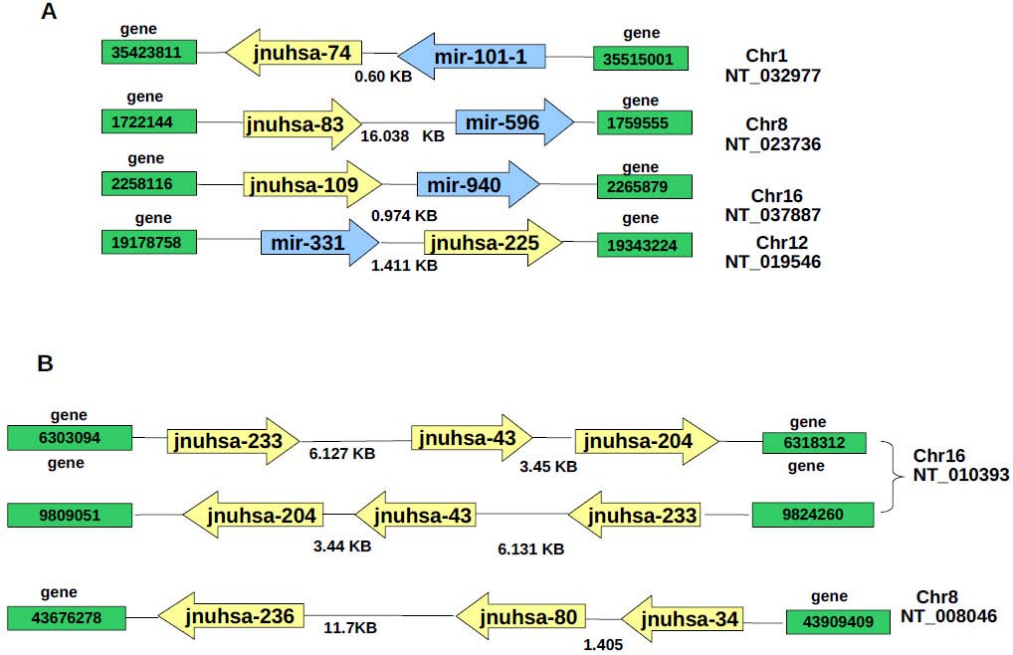
**Clustering of the novel miRNAs**. A. Novel miRNAs occurring in the vicinity of the known miRNAs. B. Novel miRNAs forming a new cluster.

In principle deep sequencing of sRNAs should generate sequences from as yet unannotated regions of the genome. The analysis of the sequences through computational pipeline (Figure 9) showed a large number of unannotated sequences that are encoded by either intergenic or intronic regions. Since miRNAs are predominantly encoded by intergenic and intronic regions, these sequences were analyzed by a set of computational tools to identify putative novel miRNAs. Since predictions are based on identifying miRNA precursors, genomic regions (70 nucleotides) surrounding the sRNA sequences were extracted (see "Methods" for details). A total of 370 (357 major + 13 minor) novel miRNAs were predicted using computational pipeline (Additional file 6). The sequences and chromosomal locations of the predicted novel miRNAs from intronic and intergenic regions are listed in the supplementary table (Additional files 7 and 8). More than 95% of the novel miRNAs showed frequency counts lower than 10. Many novel miRNAs in our dataset showed presence of 1-11 isomiRs with varying frequencies. For example, frequencies ranging from 1-280 were observed for eight isomiRs of jnuhsa-204. Presence of isomiRs certainly strengthens classification of these molecules as novel miRNAs [47]. These datasets were also analyzed using miRDeep, a published tool for miRNA analysis of deep sequencing data [48]. The number of novel miRNAs predicted by miRDeep is much less than that using the pipeline described here (Additional files 7 and 8). This may be due to stringent criteria used by miRDeep that quite often misses known miRNAs. The expression of a few novel miRNAs, missed by miRDeep but predicted by "in-house tools", was validated through real-time PCR (for precursor miRNA) or RNase protection assay (for mature miRNAs) as shown in Figure 10 and 11. Interestingly miRNAs that were present as singleton (for example- jnuhsa-4-3p, jnuhsa-93-3p) were also detected using RNase protection assay (Figure 11). We also mapped predicted novel miRNAs to clusters based on the inter-miRNA distance of less than 20 kb (Figure 12). Some of the clusters contained only novel miRNAs while many others were part of previously known clusters [72]. Based on miRDeep prediction a total of 12 novel miRNAs showed similarity in the seed region with the known miRNAs indicating that these miRNAs are likely to belong to the same family and thus may share common biology (Additional files 7 and 8).

## Discussion

Our effort to understand the microRNAome of human leukocytes through deep sequencing technology has given us several interesting observations. These include discovery of novel miRNAs and a valuable list of differentially expressed miRNAs in chronic and acute myelogenous leukemia. The diversity in the miRNA wealth was realized through the detection of large number of novel miRNAs. Our major findings are a list of expressed miRNAs (534 known and 370 novel) in leukocytes and the discovery that the expression of miRNAs may be controlled by regulating post - transcriptional events, such as manipulating the level of DICER, an enzyme involved in biosynthesis of miRNAs in tumor cells (K562). Generally, singletons in the sequenced samples are not taken into consideration, as these are likely to occur due to potential sequencing error or transcription noise [73]. Our experimental results show convincingly that such miRNAs are likely to be real and not a result of artifact of sequencing.

The known miRNAs found to be most abundant in all the four samples were members of the let-7 family, 103, 185 and 320a, which were also reported in peripheral blood mononuclear cells using "Taqman microRNA assay" [50]. Therefore our results are in agreement with previous studies based on a different methodology. There are multiple mechanisms that are likely to regulate miRNA levels similar to that of mRNAs. These include both transcriptional and post - transcriptional processes. It is generally believed that levels of miRNAs are regulated transcriptionally [74,75]. In this study an attempt was made to understand the role of different mechanisms in controlling miRNA levels by studying levels of individual miRNAs present in clusters and intronic miRNAs. It is expected that all miRNAs in a cluster belong to one transcription unit and are supposed to be synthesized as one long precursor [51,52]. Since many miRNAs of a cluster showed variable expression patterns it is likely that the levels are controlled using post - transcriptional mechanisms. Therefore, regulation of post - transcriptional processing may be a preferred mechanism, instead of transcription control in case of miRNAs present in clusters. Studies describing the role of post - transcriptional mechanism in regulating levels of a number of miRNAs have been recently published [53,76]. On the other hand our results regarding relative levels of intronic miRNA and the host mRNAs do indicate that the levels of many intronic miRNAs are regulated at transcriptional level.

The isomiRs of different miRNAs were also frequently found suggesting that these do not arise due to rare events. As 5' and 3' ends of mature miRNAs are defined by processing from DROSHA and DICER, respectively, it is likely that isomiRs with extra nucleotides at the 5' end comes from variation in DROSHA processing while with extra bases at the 3' end comes from variation in DICER processing. Another possibility is processing of mature miRNA ends by yet an unidentified end-processing enzyme in the nucleus. Whether the extra nucleotides of isomiRs affect the target recognition needs to be evaluated.

A number of differentially expressed miRNAs and their potential targets were identified by comparing miRNA and mRNA levels of normal PBMC versus that of two myeloid cancer cell lines K562 and HL60 along with computation approach. This strategy was used as degradation of many mRNAs was observed as a result of miRNA action [77]. Both K562 and HL60 have been used as reference cell lines for studying the cellular and molecular events involved in the proliferation and differentiation of chronic myeloid or acute promyelocytic leukemia, respectively. Many miRNAs that were previously reported to be involved as tumor suppressors, such as let-7 g, miR-101, 16 and 192 were found to be down regulated in the leukemia cell lines. Many of the differentially regulated miRNAs, identified in this study have been reported to be involved in various cellular processes like cell cycle (miR-192) [78], apoptosis (miR-16, -126, -98) [21,79,80] differentiation (miR-27a, -181, -342, -223) [28,81-83] DNA repair (miR-24, -210) [84,85] metastasis [86], erythroid maturation (miR-22) [87], erythropoiesis (miR-24) [88] and hematopoiesis (miR-142, -181) [89,90]; highlighting their putative role in leukemogenesis or progression. Strikingly, majority of the differentially regulated miRNAs were found to be down regulated in K562 cell line. It is possible that low level of DICER, as revealed by gene expression profiling, is responsible for an overall reduction in the miRNA population. Global down regulation of miRNAs in mouse T cells using PCR and conventional sequencing approach was reported before [91]. However, the cause of the down regulation was not clear, as mRNA profiling was not carried out. DICER has also been implicated in mouse T cell functions [92]. Our studies strongly suggest that there may be a link between DICER and miRNA levels. Many of the genes, identified in this study as the potential targets of differentially regulated miRNAs are known to be involved in cancer through their effects on cell differentiation (CDK6, LIFR), apoptosis (PIM1) or hematopoiesis (GATA2, TAL1) [93-97]. Interestingly, majority of the targets of the cancer-associated miRNAs, such as -ARHGEF, CDK6, ETV5, GATA2, PIM1, LIFR, TAL1, PPARG, RANBP17, TFRC FOXP1 LPP, NCOA2 and NR4A3 have been previously shown to be associated with leukemogenesis.

One of the features of this study is the computation pipeline that identifies novel miRNAs among sRNA sequences. This pipeline is different from other available pipelines as it has custom designed tools for identification of novel miRNAs that involve pre-processing of the sequences, an exhaustive elimination pipeline, folding and filtering to get novel predictions. The evidences supporting the predictions are identification of potential precursors by specific precursor identification tools, presence of star sequences, presence of isomiRs and in some cases experimental validation. The fact that even a single sequence read may not be a noise or error, has been validated by observing corresponding sRNA experimentally and indicates the power of our computation pipeline in identification of novel miRNAs. The total number of novel miRNAs identified from all the four samples is 370. Therefore, total number of miRNAs encoded by the human genome may be much larger than current estimate [98].

## Conclusions

In conclusion we list about 904 miRNAs that are expressed in normal and cancerous leukocytes, nearly 41% of these are novel. Our analysis shows that some of the novel miRNAs are likely to be clustered in the genome similar to many known clusters of miRNAs. Moreover, we have also identified a number of miRNAs that are differentially expressed in cancer cells studied by us. These are likely to be new markers/cause of carcinogenesis. In addition we show that miRNA levels can be regulated at post - transcriptional processing stage. Our results also show that singletons in deep sequencing reads are unlikely to be sequencing artifacts. In K562 cells low levels of miRNAs is correlated with reduced level of the enzyme DICER. In addition we have developed an improved automated computation pipeline for analysis of deep sequencing data to obtain quantitative profiles of known and novel miRNAs. Based on our observations, it is now possible to generate leukocyte specific miRNA arrays in order to study expression profiles of all miRNAs relevant to leukocytes. Similar approach can be used to generate other tissue specific miRNAome.

## Methods

### Cell lines and blood samples

The human chronic myeloid leukemia blast crisis cell line, K562 and acute promyelocytic leukemia cell line, HL60 were obtained from National Centre for Cell Sciences, Pune and maintained in RPMI 1640 and DMEM (Gibco) medium, respectively. The medium was supplemented with 10% FBS and penicillin-streptomycin and maintained at 37°C with 5% CO_2 _in incubator chamber. Buffy coat of healthy blood donors (N1 and N2) were collected from volunteers. Red cell lysis buffer (0.144M NH_4_Cl, 0.01M NH_4_HCO_3_) was added to buffy coat to lyse the remnant RBCs and pure WBC population was obtained by centrifugation at 3000 g.

### RNA Isolation and sequencing

Total RNA isolation was carried out from peripheral blood and cell lines using TRIzol^® ^Reagent (Invitrogen) as per manufacturer's instruction. RNA preparations were stored at - 80-°C till further use. Small RNA (sRNA) population was isolated by separating 10 μg of total RNA on denaturing polyacrylamide gel electrophoresis (PAGE) and cutting a portion of the gel corresponding to the size 18-30 nucleotides based standard oligonucleotide markers. Adapter (5') was ligated to sRNA population and ligated RNAs (40-60 nt) were purified by running on urea PAGE. This was followed by 3' adapter ligation and purification of adapter ligated RNAs (70-90 nt) in a similar manner. Modified sRNAs were reverse transcribed and then PCR amplified with adapter specific primers and the amplified cDNAs were finally purified on Urea PAGE to generate cDNA tag libraries for sequencing by illumina genome analyzer. The average number of sequencing reads was around 4.9 million.

### Datasets

Four sRNA sequencing data comprising of peripheral blood leukocytes of two normal individuals (N1, N2) and tumor cells K562 and HL60 were obtained from Illumina fast track sequencing services. For each sample a Sequence file and a Tag file was provided. The Tag file comprises of unique sequences with their corresponding frequency. Tag Files are generated post alignment as a summary of Sequence Files and every Sequence File has a corresponding Tag File. Tag Files are generated to give the researcher an indication of most common to most rare sequences in the dataset. The numerical frequency of each sequence in the Sequence File for gene expression gives a true indication of relative expression of sequence transcripts. The unique sequences in the Tag files contain a 3' adaptor sequence (TCGTATGCCGTCTTCTGCTTG). The amount of the 3' adapter is variable and is dependent on the length of the sRNA. Each delivered sequence is 33/35 bases in length. A part of the adaptor sequence is seen in each sequence if the sRNA is shorter than 33/35 bases. This adapter and segments of it needs to be trimmed for proper alignment to the transcriptome/genome.

### Preparation/Processing of the datasets

***i). Removal of the adaptor sequences: ***Since the sequence of the adaptor is known, a perl script was written to trim the adaptors.

***ii). Clustering and removal of redundancy after removal of the adaptor sequence: ***Although the tag file contained unique sequences, there were some, which after the removal of the adaptor were redundant. These identical sequences were represented once and their frequency was summed up.

*iii). Sequences less than 10 nucleotides in length were excluded*

***iv). Conversion into fasta format: ***The final trimmed file was then converted into fasta format where the unique header (sequence identity) retained the information of the sequences length and frequency. The sequence ID comprised of a running number along with the length and frequency of that sequence.

### Small RNA annotation

The sRNA sequences obtained were annotated against the known databases using the following protocol-

#### i). Known/annotated sequence databases

List of the databases used for the annotation/elimination pipeline were:

a) **Mature miRNAs: **from miRNA registry, release 14 (includes a total of 904 miRNAs [718 mature (major) +186 stars (minors)].

b) **ncRNAs: **from Ensembl "Homo_sapiens.NCBI36.56.ncrna.fa" (includes the precursor miRNAs and other ncRNAs like sn/sno/sca RNAs, tRNAs, rRNAs).

c) **RNA database **from the FTP site NCBI (includes rRNAs and mRNAs).

d) **The exons **were obtained from the Contig files by a self written script.

e) **Intergenic/intronic sequences: **obtained through in-house built Perl script (using the *Homo Sapiens *Contig file 29 Feb, 2008 version). These sequences served as a source for finding novel miRNAs (intergenic/intronic).

#### ii). Finding Known miRNAs

One of our objectives was to study the expression pattern of the known miRNAs. To generate the expression profile of the known miRNAs, the sRNA sequences of all the 4 samples were matched against the known miRNA sequences using BLASTN and an in house built pattern matching tool. The parameters used for BLAST were tuned to obtain maximum matches, such as the word size was set to 7 nucleotides, filtering was turned off and the number of alignments reported were increased.

#### iii). The Elimination Pipeline

An in house - built script was written to do a fast matching of the sequences with the created databases. A mismatch of up to 2 nucleotides was allowed. The pool of unmatched sequences at the end of the pipeline served as a source of novel miRNAs (Figure 1).

### Normalisation of the data/Calculation of Transcripts Parts Per Million (TPM)

Normalisation was carried out as the total number of reads from different experiments was not same and variations in the number of reads of individual miRNA can be due to sequencing depth. The number of reads of a transcript/sequence (representing a known miRNA) was divided by the total clone count of the sample and multiplied by **10**^**6**^. The total clone count is the sum of the frequencies of all the unique sequences/transcripts present in the trimmed file. The Additional file 1 contains a list of the known miRNAs found in all the 4 samples along with their respective frequencies and TPM values.

### Selecting Differentially expressed MicroRNAs

Differentially expressed miRNAs were identified by using a combination of two methods:

#### i) SAM Analysis

We performed the t-test procedure within significance analysis of microarrays (SAM) to look for differentially expressed miRNAs. SAM calculates a score for each gene on the basis of the change in expression relative to the standard deviation of all measurements.

#### ii) Fold Change

The known miRNAs in normal samples were compared to the cancer cell line. The miRNAs showing more than 2.5 fold difference as compared to both the normal cells were considered as differentially regulated. The samples with less than 10 TPM in both normal and cancer samples were ignored.

### Novel miRNA Prediction

The strategy is based on first removing all known RNAs including those derived from exonic regions and then identifying those that are derived from intronic and intergenic regions. These were then subjected to some of the *ab initio *miRNA prediction algorithms like SCFG based CID-miRNA [99] and CSHMM [100] that use stringent criteria to distinguish between real and pseudo miRNAs. Besides this we ensure high discriminative power by using the following filters:

a) Occurrence of the sequence in the stem region of the precursor

b) Presence of IsomiRs

c) Presence of minor/star sequences.

d) Taking a consensus among the prediction tools

Following is the detailed pipeline used for the prediction of novel miRNAs:

#### i) Extraction of matches from the intergenic/intronic regions of the human genome

The unmatched sequences (from the elimination pipeline) were matched to the intergenic/intronic regions. The exact matched sequences were extracted along with 70 nucleotides flanking both the ends representing potential precursor sequences.

#### ii). Folding the extended sequences and checking its location in the folded structure

The sequences were scanned for presence of potential precursor miRNA using CID-miRNA [99] and CSHMM [100] prediction tools. The folded sequences generated were then checked to see if the sRNA (putative mature miRNA obtained by sequencing) occurs in the folded putative precursor as the window scanning approach used could report a folded structure not involving the concerned sRNA. Only those hairpins were kept which contained sRNA. The next step involved locating the position of the sRNA in the hairpins. Since mature miRNAs are known to be arising from the stem portion and not the loop, only those hairpins in which the sRNAs occurred in the stem were classified as correct cases and the remaining as prediction errors. These correct cases were further tested by MiPred [101].

#### iii). Finding IsomiRs and Star sequences

A list of all the predicted correct precursor sequences was created and the sRNAs derived from common precursors were grouped into a common family. The sRNAs derived from the same precursors were kept together in a family. The most abundant member was designated as the mature miRNA. The sRNAs that differed from the representative by a few nucleotides were called IsomiRs and those that had a different, partially complementary sequence and were located in the other strand (stem of the hairpin loop) were called stars. The Additional files 7 and 8 comprise of the novel miRNAs grouped into families on the basis of sRNAs falling within the same precursor. The representative sequence was chosen on the basis of abundance. The most abundant sequence was selected as representative of the family. The scores from the 4 tools (CID-miRNA, CSHMM, miRDeep, MiPred assigned to the corresponding precursors are also listed).

#### iv). Locating the potential - novel miRNA in the genome

On the basis of the positions of the predicted novel miRNAs, their tendency to occur in a known cluster or a new cluster was checked. miRNAs located within 20 kb of the known or novel miRNA were considered as part of the same cluster.

#### v). Removal of the redundant miRNAs to get the final list of novel miRNAs

The novel miRNAs from the 4 samples (both intergenic and intronic) were pooled and the redundancy was removed to get a final set of novel miRNAs. These were given a unique name (Additional file 6). The representative miRNAs in the Additional files 7 and 8 along with their sample IDs also have these names.

### RNAse Protection Assay (RPA) for detection of mature miRNA and real time RT-PCR for precursor miRNA detection

The RPA assay for mature miRNA detection was done using *mir*Vana ™ miRNA detection kit as per supplier's instructions (Applied Biosystems). To detect the precursor miRNA, total RNA was treated with DNAase I (MBI fermentas). Reverse transcription was done using superscript^® ^III (Invitrogen) reverse transcriptase as per supplier's instructions. A list of primers used for real time RT-PCR amplification of precursors of novel miRNAs is given in Additional file 9. Real-time PCR was done with following parameters- Initial denaturation - 94°c for 2 min, denaturation- 94°c for 30 sec, annealing- 60°c for 1 min for 40 cycles using SYBR Green PCR Master Mix (Applied biosystems).

### Microarray analysis

Total RNA from normal PBMC and two myeloid leukemia cancer samples (K562 and HL60) was sent for gene expression profiling using Ocimum microarray platform (Ocimum, Hyderabad, India). The expression data for each sample was generated on Affymetrix Human Gene 1.0 ST arrays. A fold change cut-off of ± 1.5 resulted into FDR < 0.001 for both the comparisons. The quality control analysis was carried out using Affymetrix Expression Console (EC). The statistical analysis was performed using R-programming language and the biological analysis was carried out using GenowizTM software. The data obtained was normalized and genes showing more than 1.5 fold differences in the cancer cell lines as compared to normal were marked as differentially expressed genes. A total of 1856 genes were found to be up regulated and 1696 were down regulated in K562. In HL60, 1497 genes were up regulated and 1213 genes were found as downregulated (Additional file 10).

### miRNA target prediction

The most probable targets of the differentially regulated miRNAs were fished out using following two criteria - 1. Prediction by at least five of the established target prediction programs- A list of putative targets was prepared (List A) using intersection between at least five of the eleven established target prediction programs compiled by mIRecords: (DIANA-microT, MicroInspector, miRanda, MirTarget2, miTarget, NBmiRTar, PicTar, PITA, RNA22, RNAhybrid, and TargetScan/TargertScanS). 2. Inverse correlation in expression pattern between miRNA and coding genes- The putative target genes in list A was compared to the list of differentially regulated genes (showing more than 1.5 fold difference) (List B) in K562 and HL60 as obtained from expression profiling data, and only those genes that show inverse correlation to the miRNA levels were considered as most genuine putative targets of the select miRNAs.

## Availability and Requirements

The entire computational pipeline described in this paper is available at the website: http://mirna.jnu.ac.in/deep_sequencing/deep_sequencing.html. The software is also available on request.

### Accession numbers

The data discussed in this publication have been deposited in NCBI's Gene Expression Omnibus and are accessible through GEO Series accession number GSE19833 Super series: http://www.ncbi.nlm.nih.gov/geo/query/acc.cgi?acc=GSE19833

The GSE19833 Super series comprises of the following sub series:

Microarray Expression data: GSE 19789 (consists of raw data and normalized data) Deep sequencing data: GSE 19812 sequence data (consists of the raw sequences, untrimmed tag and trimmed tag files and the expression profile of the known miRNAs (Additional File 1).

## Abbreviations

miRNA: microRNA; UTR: untranslated region; RISC: RNA induced silencing complex; PTEN: phosphatase and tensin homolog; RAS: rat sarcoma viral oncogene homolog; HMGA2: high mobility group AT-hook 2; BCL2: B-cell CLL/lymphoma 2; PBMC: peripheral blood mononuclear cell; SAM: significance analysis of microarrays; FDR: false discovery rate; CML: chronic myelogenous leukemia; AML: myelogenous leukemia; ABL: c-abl oncogene 1, receptor tyrosine kinase; MEIS2: Meis homeobox 2; SMAD7: SMAD family member 7; TRIB2: tribbles homolog 2 (Drosophila); MAPK: mitogen activated protein kinase; BCL11A: B-cell CLL/lymphoma 11A (zinc finger protein); ETV4: ets variant 4; IDH1: isocitrate dehydrogenase 1 (NADP+), soluble; PPARG: peroxisome proliferator-activated receptor gamma; EIF4A2: eukaryotic translation initiation factor 4A, isoform 2; LCK: lymphocyte-specific protein tyrosine kinase; NCOA2: nuclear receptor coactivator 2; NR4A3: nuclear receptor subfamily 4, group A, member 3; PIM1: proviral integration site 1; RNASEN: ribonuclease type III, nuclear; DGCR8: DiGeorge syndrome critical region gene 8; XPO5: exportin 5; RAN: ras-related nuclear protein; PRMT5: protein arginine methyltransferase 5; FXR2: fragile X mental retardation, autosomal homolog 2; MOV10: Moloney leukemia virus 10; SNP: single nucleotide polymorphism

## Authors' contributions

AB and LK conceptualized the study. CV generated the computational pipeline, processed, normalized and annotated the data and did the novel miRNA prediction analysis. RK and HMA conducted the miRNA differential expression, miRNA cluster, microarray data and target prediction analyses and wet experiments. PS provided the RNA samples for sequencing. RG conducted SAM analyses. RK and AB wrote the manuscript. All the authors read and approved the final manuscript.

## Supplementary Material

Additional file 1 **The known miRNAs expression pattern for all the 4 samples**. The known miRNAs found in the 4 samples with their corresponding frequency and TPM values. Click here for file

Additional file 2 **miRNA cluster analysis**. Cluster analysis of the known miRNAs in Normal1, Normal2, K562 and HL60. Click here for file

Additional file 3 **Comparison of the sensitivity of the three miRNA detection methods (Deep sequencing reads, RNase Protection assay and quantitative RT-PCR)**. Fold change differences in normal PBMC versus K562 is presented for miR-27a and miR-22 using the three transcript detection methods. Click here for file

Additional file 4 List of predicted targets of differentially regulated miRNAs showing inverse correlation in microarray data in K562. Click here for file

Additional file 5 List of predicted targets of differentially regulated miRNAs showing inverse correlation in microarray data in HL60. Click here for file

Additional file 6 **List of the predicted 370 novel miRNAs**. The precursor and mature sequences of the total novel miRNAs are given as text file. When the relative abundances clearly indicate which the predominantly expressed miRNA is, the mature sequences are assigned names of the form, jnuhsa-233 (the predominant product) and jnuhsa-233* (from the opposite arm of the precursor). When the data are not sufficient to determine which sequence is the predominant one, names like jnuhsa-118-5p (from the 5' arm) and jnuhsa-118-3p (from the 3' arm) have been used. Click here for file

Additional file 7 **Details of the intronic novel miRNAs of the 4 samples**. List of the novel miRNAs predicted sample wise, with the information on their frequency, isomiRs, chromosomal location and prediction scores from CID-miRNA, CSHMM, miRDeep, MiPred. Click here for file

Additional file 8 **Details of the intergenic novel miRNAs of the 4 samples**. List of the novel miRNAs predicted sample wise, with the information on their frequency, isomiRs, chromosomal location and prediction scores from CID-miRNA, CSHMM, miRDeep, MiPred. Click here for file

Additional file 9 A list of primers used for amplification of precursors of novel miRNAs. Click here for file

Additional file 10 The normalized expression data along with a list of differentially regulated genes (> 1.5 fold) obtained using Ocimum microarray platform for K562 and HL60. Click here for file
