# Supplementary figures and images for: Analysis of microRNA transcriptome by deep sequencing of small RNA libraries of peripheral blood
Source: BMC Genomics. 2010 May 7;11:288. doi: 10.1186/1471-2164-11-288 (PMC2885365; doi:10.1186/1471-2164-11-288)

## Slide 1
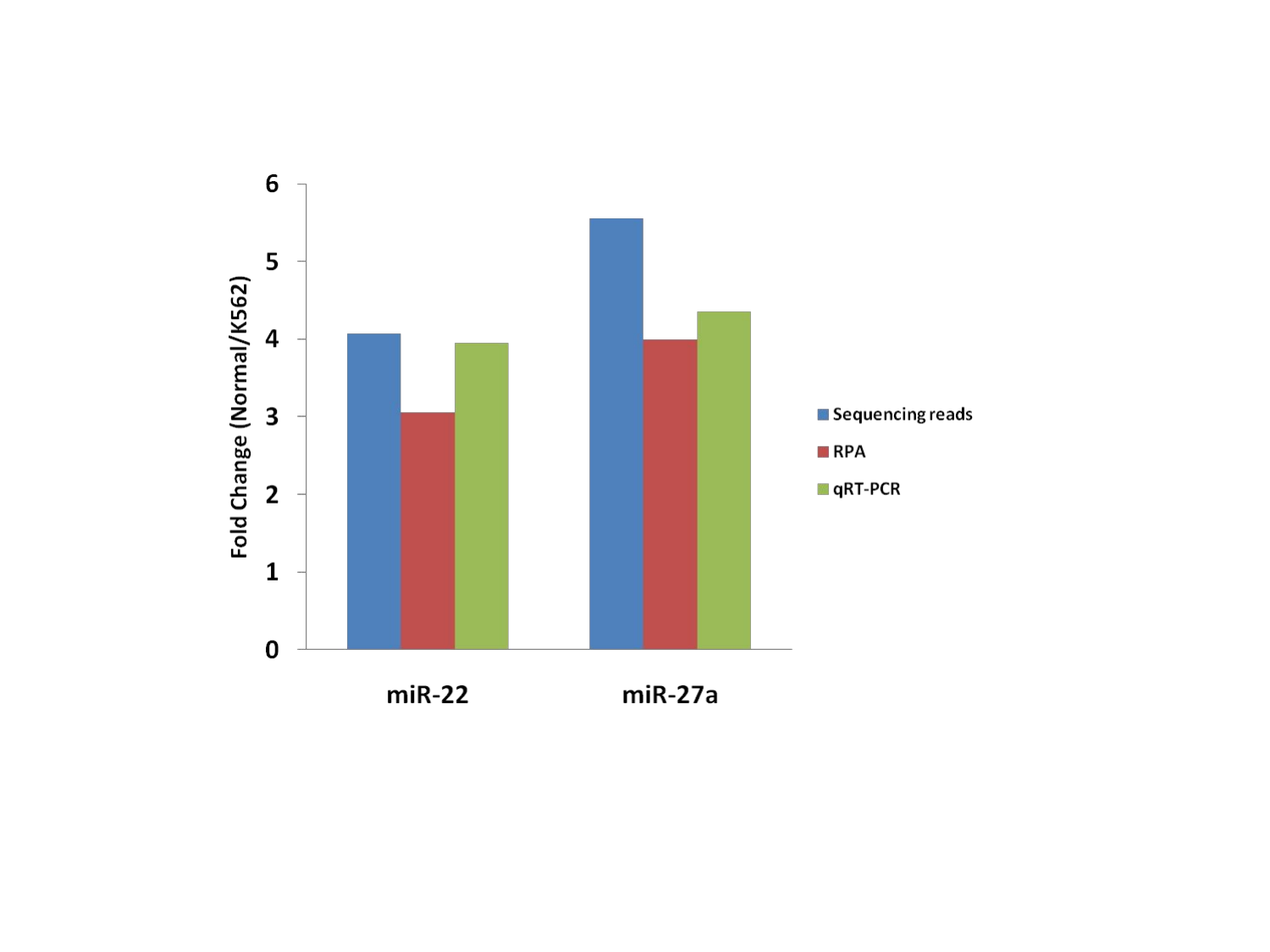

Supplement: Additional file 3 — Comparison of the sensitivity of the three miRNA detection methods (Deep sequencing reads, RNase Protection assay and quantitative RT-PCR). Fold change differences in normal PBMC versus K562 is presented for miR-27a and miR-22 using the three transcript detection methods. [file 1471-2164-11-288-S3.PPT]
