# Supplementary material for: Analysis of microRNA transcriptome by deep sequencing of small RNA libraries of peripheral blood
Source: BMC Genomics. 2010 May 7;11:288. doi: 10.1186/1471-2164-11-288 (PMC2885365; doi:10.1186/1471-2164-11-288)
Supplement: Additional file 4 — List of predicted targets of differentially regulated miRNAs showing inverse correlation in microarray data in K562. [file 1471-2164-11-288-S4.PDF]

List of predicted targets of differentially regulated miRNAs showing inverse correlation in microarray data in K562.

let-7g	let-7i	miR-1	miR-16	miR-21	miR-22	miR-24	miR-27b	miR-27a	miR-30e	miR-186	miR-192	miR-339-3p	miR-101	miR-106b	miR-142-5p	miR-146b-5p	miR-342-3p	miR-486-3p	miR-504
IGF2BP1	MEIS2	CLCN3	SPRED1	NFIB	ARHGEF12	HIC2	TEAD1	RELN	ENOX2	KIAA1324L	PKP4	TUBB	EZH2	FAM57A	ZFPM2	STRBP	WDR77	ZC3H12A	RNF38
SCD	IGF2BP3	BET1	DOLPP1	ZNF367	PDIK1L	SLC19A2	GALNT7	NEDD4	NFIB	PSPH	MIPOL1	METT5D1	ABCC5	ZFPM2	EGLN3	PTGFRN	SETD8	PLEKHO2	BACH2
ABCC5	CDC25A	PTPLAD1	PCDH9	CDC25A	CPEB1	PLOD2	ANK1	SGMS1	GALNT2	SETD8		BBS5	DCBLD2	SSX2IP	SGMS1	BIVM	TBX18	SH3KBP1	TP53INP1
LGR4	SCD	NETO2	WEE1	SCML2	YARS	MTHFR	SATB2	ANK1	VAT1	C6orf167		CDC25A	SLC19A2	ETV1	IGF2BP3	SORT1	ZAK	KIF21B	TNFAIP2
CDC25A	C15orf41	HSPD1	VEGFA		WASF1	CMTM4	TMUB1	TMUB1	GTF2H1	PHF6		ADAMTS14	CLCN3	KIF23	PDIK1L	PSMD3		PITPNC1	CD86
LIN28B	LIN28B	SPRED1	ADAMTS3		CPEB1	WHSC1	CLCN3	TEAD1	DOLPP1	PFKFB4		WDR5	STMN1	EGLN3	BAI3	HIC2		CPEB2	CR1
HIC2	HIC2	BPNT1	HSPA4L		NET1	DCBLD2	NEDD4	GALNT7	PTGFRN	CALB1		LMNB2	LRCH2	NEDD4L	DIAPH3	ADAMTS3		PLCB1	PAPD5
BZW2	BZW2	GNPNAT1	KIF23		MTHFR	DHFR	E2F7	HSDL1	GALNT7	EIF2S2		ADAMTS14	DDIT4	MYO1D	HS2ST1			SIRPA	SBF2
STRBP	C6orf211	CPEB1	HIGD1A		TYRO3	AARS	SGMS1	GATA2	DDIT4	RBM9		WHSC1	PHF6	ZNF367	LRP12			AXUD1	FOXP1
RAVER2	DCLRE1B	WDR61	NEBL		IPO7	CLCN3	GATA2	ARRDC4	ADAMTS3	ACVR2B			SPRED1	LACE1	ETV1			PLEK	APOL6
RDH10	LGR4	MIPOL1	KIF5A		SATB2		ARRDC4	E2F7	SSX2IP	NASP			SOCS2	PLS1	CIT			LRP1	
RPUSD3	STRBP	PHF6	ACVR2B				HSDL1	NEDD4	NEDD4L	IPO5			TAL1	NAPEPLD				CYFIP2	
NAPEPLD	RPUSD3	C7orf58	DIXDC1				ORC5L	PHB	VKORC1L1	DPH2			SMARCA4	POLQ				ABI3	
CDC25A	ARRDC4	HOXB4	CHEK1				PHB	NEK2	GRB10	NASP			CDK6	PTGFRN				VAMP2	
ARRDC4	ZNF280B	PDIK1L	SLC13A3				RELN	PDIA5	CMTM4				ACVR2B	FAM40B				KLF12	
DCLRE1B	AP1S1	MAP1A	PIM1				BAG2	WEE1	NEDD4				MAGI1	PHF6				DIAPH2	
C6orf211	RDX	HSPD1	STXBP1				HOXB3	XPO1	CCNE2				BZW2	SGMS1				NAMPT	
C15orf41	GALE	SLC31A1	CCNE1				LIFR	BAG2	LMLN				STMN1	NUP35					
RDX	CPEB1	HMGCR	RNF217				GLT25D2	CLCN3	ZNF280B				MEIS2	CENPO					
GALE	IGF2BP2	CDK6	DIXDC1				FAM98A	GNG12	AMOTL1				ETV5	HOXB4					
CPEB1	CLDN12	PGM2	SPRY4				PPME1	SLC7A11	MEIS2				VKORC1L1	TET1					
BCL2L1	IGF2BP2	ASH2L	LMAN2L				NEDD4	AK2	SEH1L					SPRY4					
IGF2BP2	IGF2BP1	SLC25A1	MOBKL2B				PDIA5	FAM98A	SNX33					TAL1					
IGF2BP3	GALNT2		DCBLD2				SLC7A11	SATB2	PHF6					WEE1					
CLDN12	ABCC5		CARM1				MEIS2	HMGCS1	ME1					ZFYVE9					
			PCDH9				CIT	MEIS2	MYBL2					KIF5A					
			CDC25A					CIT	HERC2					TRIP10					
			E2F7						LIN28B					LDLR					
			RELN						CIT					FNBP1L					
			SLC13A3						EXTL2										
			CHAC1						HIC2										
			CDC25A						SATB2										
			PDIK1L						RAI14										
			LRP6																
			CCNE1																
			PDIA6																
			PCMT1																
			CLDN12																
			SATB2																

The targets identification is based on inverse correlation of miRNA and mRNA expression data and at least five *in silico* prediction tools as described in “Methods”.
