# Supplementary material for: Analysis of microRNA transcriptome by deep sequencing of small RNA libraries of peripheral blood
Source: BMC Genomics. 2010 May 7;11:288. doi: 10.1186/1471-2164-11-288 (PMC2885365; doi:10.1186/1471-2164-11-288)
Supplement: Additional file 5 — List of predicted targets of differentially regulated miRNAs showing inverse correlation in microarray data in HL60. [file 1471-2164-11-288-S5.PDF]

List of predicted targets of differentially regulated miRNAs showing inverse correlation in microarray data in HL60.

miR-1	miR-7	miR-21	miR-22	miR-27b	miR-30e	miR-101	miR-106b	miR-146b-5p	miR-152	miR-194	let-7a	let-7b	let-7c	let-7d	miR-25	miR-98	miR-99a	miR-181a	miR-181b	miR-221	miR-425
BET1	DACH1	JAG1	TRUB1	RPGRIP1L	ERLIN1	EZH2	NUP35	DTNA	RALBP1	TFRC	SLC35D2	SLC35D2	STK40	STK40	SGPP1	GCNT4	TRIB2	TOX	NR4A3	CDKN1B	PAG1
NETO2	TFRC	PHF14	WASF1	FAM98A	VAT1	DCBLD2	M6PR	STRBP	GTF2H1	MEIS2	TMEM2	DUSP16	DUSP16	TMEM2	CPEB4	STK40	BAZ2A	PRKCE	LPP	FOS	CT1
HSPD1	HELLS	SPRY2	SATB2	EBF3	GTF2H1	EDEM3	RBL1	DTNA	DPP3		STK40	PPP1R16B	TMEM2	GCNT4	BTG2	KLHL24	CTDSPL	ZFP36L1	NCOA2	TOX	SLC37A1
SPRED1	LRRC59	SCML2	IPO7	ENAH	PTGFRN	GPR85	VLDLR	BIVM	TOMM70A		DUSP16	GPX7	GCNT4	SLC35D2	ARRDC3	CD200R1		NR4A3	PRKCE	GARNL1	LRIG1
RPE	NXT2	FAM46A		BAG2	GALNT7	SLC19A2	WEE1	DTNA	B4GALT2		GCNT4	LRIG1	GPX7	LRIG1	KLF4	SLAMF6		MBOAT2	MBNL2	KIF16B	WWC3
SLC25A1	GALNT7	SPG20		C5orf13	GRB10	FBN2	SRPK1	PTGFRN	SMS		KIAA1539	TRIB2	TRIB2	SLAMF6	JOSD1	CD200R1		SYNE1	ZFP36L1	CPNE8	ARMCX3
DACH1		CDC25A		PPARG	CMTM4	STMN1	CENPO		TFRC		TRIB2	GCNT4	SLC35D2	CD200R1	DOCK9	DUSP16		CPEB4	NMT2	ETS1	AFF4
PGM2		ZNF367		PHB	TSGA14	CCDC18	PHF6		ZADH2		KLHL24	TMEM2	SNN	SYT11	HERPUD2	KIAA1539		FBXO33	SYNE1	UNC84B	RICTOR
CDK6		BCL11A		ORC5L	CCNE2	SELI	PTGFRN		LRP8		CPEB2	STK40	PPP1R16B	CD200R1	PLEKHA1	CPEB2		ZBTB4	HECA	GARNL1	TMEM56
HSPD1				DTNA	DOCK7	PHF6	USP31		BCL11A		CD200R1	SLAMF6	CD200R1	CPEB2	CPEB2	SNN		ADM	ZBTB4	TRPS1	C12orf23
BCL11A				MFSD2	SLC39A11	SPRED1	POLQ		NR2C2AP		SLAMF6	CD200R1	SYT11	KIAA1539	KLF2	ITGB3		MAP3K3	TOX	KIAA1370	KLRD1
PHF6				ZADH2	EDEM3	APP	LASS6		DNMT1		RAB11FIP4	SYT11	SLAMF6	SNN	ADM	IGF2BP2		ATP2B1	ATP2B1		BEX4
WDR61				SLC39A11	MEIS2	CDK6	LACE1		ERLIN1		CD200R1	KIAA1539	KIAA1539	CCR7	EGR2	EGR3		ATP1B1	EIF4A2		ANXA2
GPR85				RET	PRICKLE1	ACCN2	ZNF367		AKAP1		SYT11	RAB11FIP4	RAB11FIP4	SLC4A4	GRAMD3	SLC30A4		B4GALT1	CBLB		ITGAX
GNPNAT1				DTNA	SEH1L	BZW2	SASH1				GPX7	CPEB2	CPEB2	UTRN	FCHO2	UTRN		ATP1B1	ATP1B1		RIT1
SLC44A1				ATXN10	PHF6	MEIS2	KIF23				SNN	CD200R1	CD200R1	MYO1F	ADAM19	PPP1R16B		KIAA1128	TGFBI		LITAF
BPNT1				EDEM3	MYBL2	EZH2	SACS				SLC4A4	KLHL24	KLHL24	ARID3B	SNN	LRIG1		RIN2	KLHL5		CTSS
PTPLAD1				SATB2	SATB2	ETV5	NAPEPLD				ITGB3	EGR3	SLC30A4	EGR3	PRKCE	SLC35D2		CTDSPL	ARRDC3		CSGALNA
				GALNT7	VKORC1L1	VKORC1L1					CCR7	EPHA4	MYO1F	RASGRP1	HIVEP1	IGF2BP2		KLHL5	RIN2		
				NXT2							IGF2BP2	UTRN	ITGB3	RAB11FIP4	CD69	ARID3B		CACNA2D2	FBXO33		
				MEIS2							MYO1F	SLC30A4	CCR7	PPP1R16B	S1PR1	EPHA4		ARRDC3	MAP3K3		
											SLC30A4	MYO1F	SLC4A4		SMAD7	TRIB2		ARL5B	MBNL2		
											UTRN	IGF2BP2	IGF2BP2		BAZ2A	RAB11FIP4		JAZF1	FOS		
											LRIG1	ITGB3	ARID3B		TOB1			MAP3K3	CPEB4		
											PPP1R16B	SLC4A4	EGR3					TGFBI	SMAD7		
											EGR3	SNN	EPHA4					NR4A3	B4GALT1		
											EPHA4	MAP3K3	LRIG1					MBNL2			
											RASGRP1	CCR7	UTRN					ATP2B1			
											ARID3B							CBLB			
											IGF2BP2							C5orf41			
																		BAZ2A			
																		PDK4			
																		SMAD7			
																		CTDSPL			
																		SNN			
																		UBL3			
																		SLC2A3			
																		IGF2BP2			
																		NCOA2			
																		LPP			
																		EPHA4			
																		EGR3			
																		DUSP5			
																		FOS			
																		ETS1			
																		NMT2			
																		HECA			

The targets identification is based on inverse correlation of miRNA and mRNA expression data and at least five *in silico* prediction tools as described in "Methods".
