# Supplementary material for: Analysis of microRNA transcriptome by deep sequencing of small RNA libraries of peripheral blood
Source: BMC Genomics. 2010 May 7;11:288. doi: 10.1186/1471-2164-11-288 (PMC2885365; doi:10.1186/1471-2164-11-288)
Supplement: Additional file 9 — A list of primers used for amplification of precursors of novel miRNAs. [file 1471-2164-11-288-S9.DOC]

|  | **MicroRNA** | **5’Primer** | **3’ Primer** |
| --- | --- | --- | --- |
| 1. | Jnuhsa91 | CCACTGAGCTGGACCACTTAGC | CCAGCGAGACAGAACCATTCAC |
| 2. | Jnuhsa204 | GGGCGGAGCTTCCAGACG | GGAAGCTCCGCCCCACGC |
| 3. | Jnuhsa75 | GGCTGGTGGCGAGTTCCG | GGCAGGCGAGAATTCTACCACTG |
| 4. | Jnuhsa93 | GATGATAAGTTATGGGGCTTCTG | GAATTCAGTTTGGGGGAGTTC |
| 5. | Jnuhsa245 | CCTGGGAGGTGTGATATCATG | TCCCAGGAACCACAATATCAC |
